# Exploring signatures of positive selection in pigmentation candidate genes in populations of East Asian ancestry

**DOI:** 10.1186/1471-2148-13-150

**Published:** 2013-07-12

**Authors:** Jessica L Hider, Rachel M Gittelman, Tapan Shah, Melissa Edwards, Arnold Rosenbloom, Joshua M Akey, Esteban J Parra

**Affiliations:** 1 Department of Anthropology, University of Toronto at Mississauga, Mississauga, Ontario, Canada; 2 Department of Genome Sciences, University of Washington, Seattle, Washington, USA; 3 Department of Mathematical and Computational Sciences, University of Toronto at Mississauga, Mississauga, Ontario, Canada

## Abstract

**Background:**

Currently, there is very limited knowledge about the genes involved in normal pigmentation variation in East Asian populations. We carried out a genome-wide scan of signatures of positive selection using the 1000 Genomes Phase I dataset, in order to identify pigmentation genes showing putative signatures of selective sweeps in East Asia. We applied a broad range of methods to detect signatures of selection including: 1) Tests designed to identify deviations of the Site Frequency Spectrum (SFS) from neutral expectations (Tajima’s D, Fay and Wu’s H and Fu and Li’s D* and F*), 2) Tests focused on the identification of high-frequency haplotypes with extended linkage disequilibrium (iHS and Rsb) and 3) Tests based on genetic differentiation between populations (LSBL). Based on the results obtained from a genome wide analysis of 25 kb windows, we constructed an empirical distribution for each statistic across all windows, and identified pigmentation genes that are outliers in the distribution.

**Results:**

Our tests identified twenty genes that are relevant for pigmentation biology. Of these, eight genes (*ATRN, EDAR, KLHL7, MITF, OCA2, TH, TMEM33* and *TRPM1,*) were extreme outliers (top 0.1% of the empirical distribution) for at least one statistic, and twelve genes (*ADAM17, BNC2, CTSD, DCT, EGFR, LYST, MC1R, MLPH, OPRM1, PDIA6, PMEL (SILV)* and *TYRP1*) were in the top 1% of the empirical distribution for at least one statistic. Additionally, eight of these genes (*BNC2, EGFR, LYST, MC1R, OCA2, OPRM1, PMEL (SILV)* and *TYRP1*) have been associated with pigmentary traits in association studies.

**Conclusions:**

We identified a number of putative pigmentation genes showing extremely unusual patterns of genetic variation in East Asia. Most of these genes are outliers for different tests and/or different populations, and have already been described in previous scans for positive selection, providing strong support to the hypothesis that recent selective sweeps left a signature in these regions. However, it will be necessary to carry out association and functional studies to demonstrate the implication of these genes in normal pigmentation variation.

## Background

The major out of Africa migrations of anatomically modern humans took place within the last 60,000-50,000 years [1]. As a result of these migrations, humans encountered novel environments, with varying climates, pathogens and foods and adapted to these new conditions via natural selection. One of the climatic factors showing clear geographic patterns is ultraviolet (UV) radiation, which is more intense and shows less seasonality in equatorial and tropical areas than in high latitude regions [2]. Skin pigmentation, which is primarily determined by the amount, type and distribution of cutaneous melanin, also shows a clear latitudinal gradient, and this has been explained as the result of natural selection [3-8]. In agreement with this hypothesis, genome-wide scans have shown that many genes involved in the pigmentation pathway show signatures of positive selection [9-21]. Interestingly, most of the putative selection signatures have been identified in European and East Asian populations, indicating that the majority of the selective sweeps took place after the out-of-Africa migration of modern humans. Although some of the pigmentation genes show signatures of selection that are shared between European and East Asian populations (e.g. *KITLG*) [12,18] many genes show positive selection signals in only one population (e.g. *SLC24A5* and *SLC45A2* in Europe, *DCT* in East Asia) or independent signals in European and East Asian groups (*OCA2*). These findings support an evolutionary model in which the most important changes in pigmentary traits occurred after the migration out-of-Africa and the separation of the lineages that gave rise to contemporary European and East Asian populations [5,12,22-25].

Most of the surveys of signatures of selection published to date have been based on data from the HapMap project or the Human Genome Diversity Project (HGDP), which primarily captured common genetic variants. The recent availability of the 1000 Genomes project Phase I data, which includes full genome sequences (based on a combination of low-coverage whole genome sequencing and targeted deep exome sequencing) for more than 1,000 individuals from 14 populations, has opened new opportunities to study genetic variation in our species [26]. In particular, the improved representation of rare variants and the decreased bias in variant detection would be expected to increase the power of some of the tests used to identify selective sweeps. In this study, we applied a range of genome-wide methods to detect signatures of selection in the 1000 Genomes Phase I dataset. The methods employed include: 1) Tests designed to identify deviations of the Site Frequency Spectrum (SFS) from neutral expectations (Tajima’s D, Fay and Wu’s H and Fu and Li’s D* and Fu’s F*), 2) Tests focused on the identification of high-frequency haplotypes with extended Linkage Disequilibrium (LD) (iHS and Rsb) and 3) Tests based on genetic differentiation between populations (LSBL). The main goal of the study was to identify pigmentation genes that have been the target of positive selection in East Asia. To date, the overwhelming majority of the genetic association studies focused on pigmentary traits have been carried out in European populations and as a result the last decade has brought a much better understanding of the genetic basis of normal pigmentation variation in this group [6,12,27,28]. In contrast to the long list of genes that have been associated with pigmentary traits in European populations, there is very limited knowledge concerning the genes involved in pigmentary traits in East Asia. Notable exceptions are the genes *OCA2* and *MC1R*, which harbor non-synonymous mutations, rs1800414 (His615Arg) in OCA2 and rs885479 (Arg163Gln) in MC1R, that have been associated with skin pigmentation in East Asian populations [23,29,30]. These polymorphisms are present in high frequency in East Asian populations, and are absent or present at low frequencies in European and African populations, suggesting again that there has been convergent evolution towards depigmentation in Europe and East Asia. Additional research efforts in East Asian populations, or admixed populations showing a substantial East Asian contribution [31], will be necessary in order to elucidate the genetic architecture of pigmentation in East Asian populations, and more generally, the evolutionary events responsible for the pigmentary changes that took place after the out-of-Africa migration of modern humans. By identifying pigmentation genes showing putative signatures of selective sweeps in East Asia, we will be able to prioritize a list of genes for subsequent association studies in East Asian samples characterized with quantitative methods (e.g. skin reflectometry).

## Methods

### Samples

All the statistical analyses were completed using the 1,000 Genomes Phase 1 data, which includes approximately 38 million Single Nucleotide Polymorphisms (SNPs) [26]. Indels were excluded from all the analyses. The 1000 genomes data set includes samples Japanese from Tokio (JPT), Han Chinese from Beijing (CHB) and Southern Han Chinese (CHS).

### Statistics used to identify putative signatures of positive selection

#### 1-Statistics based on the Site Frequency Spectrum (SFS)

These statistics compare different estimators of the population mutation rate ϴ = 4Nμ, which have the same expectation under neutrality.

Tajima’s D [32]. This test compares estimates of ϴ derived from the average number of pairwise differences (π) and the number of segregating sites (S).

Fu and Li’s D* [33]. This test compares estimates of ϴ derived from the number of segregating sites (S) and the number of singleton mutations (η_s_, alleles appearing only once in the sample).

Fu’s F* [34]. This test compares estimates of ϴ derived from the average number of pairwise differences (π) and the number of singleton mutations (η_s_).

For these three tests, negative values indicate an excess of rare polymorphisms, and positive values an excess of intermediate-frequency alleles with respect to neutral expectations.

Fay and Wu’s H [35]. In contrast to the previous three tests, H requires information on allele state (ancestral vs. derived). This test compares estimates of ϴ derived from the average number of pairwise differences (π) with another estimate derived from the frequency of derived alleles at segregating sites (ϴ_H_). H is negative when derived alleles are found at high frequency, with respect to neutral expectations.

These four statistics were estimated for non-overlapping 25 kilobase windows, using a Python script. The statistics were calculated independently in three East Asian samples from the 1000 Genomes Phase 1 panel: Han Chinese from Beijing (CHB) (97), Southern Han Chinese (CHS) (100) and Japanese from Tokyo (JPT) (89). Variants that did not pass the 1000 genomes project filtering metrics were masked, as well as any variants in which the ancestral allele could not be determined. Windows with less than 10 markers were excluded from further analyses.

#### 2-Tests based on genetic differentiation

We estimated genetic differentiation using the Locus Specific Branch Length (LSBL), as described in Shriver et al., 2004 [36]. In this case, we focused on the identification of regions with high East Asian LSBL values, indicating strong differentiation between East Asia and Europe/Africa. For these analyses, we used the combined 1000 Genomes Phase I East Asian (Han Chinese from Beijing, Southern Han Chinese and Japanese from Tokyo, N = 286), European (Tuscans from Italy, British, Finnish, Iberians, and Utah residents with Western European ancestry, N = 379) and African (Yoruba from Nigeria and Luhya from Kenya, N = 185) samples. East Asian LSBL values were estimated from the East Asian-African, East Asian-European and African-European pairwise F_ST_ distances for each locus using the formula LSBL(Eas) = (Eas-Eur F_ST_ + Eas-Afr F_ST_ –Afr-Eur F_ST_)/2. F_ST_ values were calculated with the program VCFTOOLS using Weir and Cockerham (1984) unbiased estimator [37]. Negative F_ST_ values were converted to zero. Using a combination of shell and python scripts, we created non-overlapping windows of 25 kilobases, and reported for each window the maximum LSBL. Windows with less than 10 markers were excluded from further analyses.

#### 3-Long-range haplotype tests

We employed two approaches based on haplotype diversity. For these tests, we restricted the analyses to markers with minor allele frequencies equal or higher than 5%. The statistics are based on the combined 1000 Genomes Phase I East Asian samples (iHS test), and the combined 1000 Genomes Phase I East Asian, European and African samples (Rsb tests). More details about the statistics are described below.

##### iHS (Integrated Haplotype Score)

iHS compares integrated EHH (Extended Haplotype Homozygosity) values between alleles at a given SNP. EHH quantifies the breakdown of LD at increasing distances from each allele (ancestral or derived). Large negative iHS values are indicative of unusually long haplotypes carrying the derived allele and large positive values are associated with long haplotypes carrying the ancestral allele [38]. iHS values were estimated using the program rehh [39].

##### Rsb

Rsb is a standardized ratio of iES (Integrated EHHS) from two populations. iES integrates the area under the curve of site-specific EHH (EHHS) [11]. Extreme values of Rsb indicate slower haplotype homozygosity decay in one population versus another. This test was designed to identify potential sweeps that have occurred only in one population. Given that we are primarily interested in identifying sweeps that are specific to East Asian populations, we focused on the comparison between East Asian and European populations, and East Asian and African populations. In this particular situation, extreme positive values of Rsb will indicate longer haplotypes in East Asian populations than in European or African populations. Rsb(Eas-Eur) and Rsb(Eas-Afr) were estimated using the program rehh [39].

After obtaining the iHS, Rsb(Eas-Eur), and Rsb(Eas-Afr) statistics for each locus, we used a combination of shell and python scripts to report the results for non-overlapping windows of 25 kilobases, indicating for each window the maximum absolute value of iHS (or the maximum value of Rsb). Windows with less than 10 markers were excluded from further analyses.

### Construction of empirical distribution of p-values based on results for 25 kb windows and identification of putative pigmentation genes under positive selection

Based on the results obtained in the analyses of 25 kb windows (e.g. values obtained for each of the SFS statistics, and maximum values for LSBL, iHS and Rsb, see above for additional information), we sorted the windows in descending order based on the values of the relevant statistics, and identified pigmentation genes that are outliers in the empirical distribution (top 0.1% or 1% of the distribution), following the approach detailed below:

#### 1-Identification of extreme outliers with empirical p-values < 0.001 and annotation of the relevant windows using the DAVID database

We used the ENSEMBL genome browser (http://useast.ensembl.org/index.html) to identify genes overlapping with the top 0.1% of the 25 kb windows for each statistic. These genes were then annotated using the DAVID database (Database for Annotation, Visualization and Integrated Discovery) [40] in order to identify genes involved in the pigmentation pathway. Briefly, we used the ENSEMBL gene IDs retrieved from the ENSEMBL genome browser as input to perform a functional annotation of each gene using DAVID Functional Annotation Tool. This tool provides different types of annotations for each gene, including annotations based on functional categories, gene ontology (e.g. GOTERM, PANTHER), pathways (e.g. BIOCARTA, KEGG_PATHWAY), protein domains (e.g. INTERPRO) and protein interactions.

#### 2-Identification of known pigmentation genes with empirical p-values < 0.01

We prepared a list of known pigmentation genes that 1) Have been associated with pigmentary traits in association studies or 2) Have been reported as outliers in previous scans of positive selection in human populations. The list included the following genes:

*ADAM17, ADAMTS20, AP3D1, ASIP, ATRN, BLOC1S6 (PLDN), BNC2, CTSD, DCT, DRD2, DTNBP1, EDAR, EDN2, EGFR, HPS1, IRF4, KIT, KITLG, LYST, MATP (SLC45A2), MC1R, MITF, MLPH, MYO5A, MYO7A, OCA2/HERC2, OPRM1, PAX3, PDIA6, PMEL (SILV), POMC, PPARD, RAB27A, RAD50, RGS19, SLC24A4, SLC24A5, TYR, TYRP1, TP53BP1, TRPM1* and *TPCN2*.

We retrieved the results of the 8 statistics analyzed in this study for all the 25 Kb windows overlapping the aforementioned genes, and identified the genes with windows in the top 1% of the empirical distributions.

Therefore, all the genes reported in the Results and Discussion section are outliers that show statistics in the top 1% of the empirical distribution, and in some cases, the top 0.1% of the empirical distribution.

## Results and discussion

We carried out genome-wide scans for signatures of selection in East Asians, with a major focus on the identification of pigmentation genes that have been under positive selection in this population group. We used three types of statistics: Statistics based on the Site Frequency Spectrum (SFS) (D, D*, F* and H), statistics based on genetic differentiation (LSBL) and long-range haplotype tests (iHS and Rsb) (See the Materials and Methods section for more details about each statistic). Importantly, these statistics are based on different characteristics of the data and are powered to identify different types of selective sweeps. For example, tests based on the SFS are primarily powered to identify older selective events and recently completed sweeps, whereas long-range haplotype tests are more useful to identify more recent events (<30,000 years ago) and incomplete or partial sweeps [41]. All statistical analyses were based on the 1,000 Genomes Phase 1 reference samples, which include approximately 38 million SNPs. For the statistical analyses, we created non-overlapping windows of 25 kb, which were used to construct an empirical distribution for each statistic. We identified genes located within the top 0.1% of the windows for each statistic and these genes were then annotated using the DAVID database in order to select genes that may potentially be involved in the pigmentation pathway. In addition to these extreme outliers, we also explored if a list of genes that have been previously associated with pigmentary traits in association studies or reported as outliers in previous scans of positive selection in human populations were located in the top 1% of the empirical distributions.

The top 0.1% 25Kb windows identified for the different statistics (957 windows) are depicted in Additional file 1: Table S1, and the basic annotations for the genes retrieved using the DAVID database (422 genes) are provided as Additional file 2: Table S2. Many of the genes reported to harbor signatures of positive selection in previous studies including East Asian populations, based on a wide range of methods, such as the Composite of Multiple Signals (CMS) test [42], the XP-EHH test [15,17] the iHS test [17,43], the LRH test [43], the Rsb test [11] and the XP-CLR test [20] are also outliers in our study. Overall, 79 genes described in these studies were also identified in our analyses, and these genes are highlighted in red in Additional file 2: Table S2. It is important to note that among the genes reported in Additional file 2: Table S2, many are located on the same genomic regions. In fact, several genomic regions are characterized by the presence of large numbers of outlier windows, such as the *EDAR* region on chromosome 2, or a genomic region on chromosome 17 characterized by extreme values for several SFS statistics (Additional file 1: Table S1 and Additional file 2: Table S2). Presumably, these are genomic regions that have been under strong and relatively recent positive selection, and the selective sweeps left a strong signature in these regions, encompassing several genes. Further analyses would be needed to determine which genes were targets of positive selection in these regions. Our primary goal in this study has been to identify pigmentation genes showing putative signals of positive selection.

Table 1 shows the list of outlier genes that are relevant for pigmentation biology. There are 20 genes in the list. Of these, 8 genes (*ATRN, EDAR, KLHL7, MITF, OCA2, TH, TMEM33* and *TRPM1,*) were extreme outliers (top 0.1% of the empirical distribution) for at least one statistic, and 12 genes (*ADAM17, BNC2, CTSD, DCT, EGFR, LYST, MC1R, MLPH, OPRM1, PDIA6, PMEL (SILV)* and *TYRP1*) were in the top 1% of the empirical distribution for at least one statistic. Most of the genes are outliers for more than one statistic, and show multiple significant windows. It is important to note that, with the exception of *TH*, *KLHL7* and *CTSD*, these genes have already been described in genome-wide scans of signatures of selection in previous studies [12-17,20,21,38,44-49], lending strong support to the hypothesis that positive selection has substantially shaped the patterns of variation of these genes. Additionally, eight of these genes (*BNC2, EGFR, LYST, MC1R, OCA2, OPRM1, PMEL (SILV)* and *TYRP1*) have been associated with pigmentary traits in association studies. The *OCA2* gene is of particular interest, because different haplotypes are associated with pigmentary traits in Europeans and East Asians. Variants located in the nearby *HERC2* gene, which affect the transcription of the *OCA2* gene, are strongly associated with blue eye color in European populations [50-55]. Another non-synonymous variant, which is common in East Asian populations but absent or very rare in Europe, has been associated with skin pigmentation in East Asia [23,29]. Several polymorphisms in the *MC1R* gene show a strong association with red hair/fair skin in European populations (Asp84Glu, Arg151Cys, Arg160Trp and Asp294His), and other variants also show a weak association with these traits (Val60Leu, Val92Met and Arg163Gln) [56]. Interestingly, the derived 163Gln allele, which is present in very high frequencies in East Asian populations (>60%), but very low frequencies in European and African populations, has been recently associated with lighter skin in an East Asian sample [30]. Polymorphisms in the *TYRP1* gene have been associated with hair and iris color in European populations [57], and a non-synonymous mutation that is found only in Oceania was recently associated with blond hair in Melanesians [58]. Frudakis et al. [59] reported association of haplotypes in the *PMEL (SILV)* gene with iris color. The *BCN2* gene has been associated with skin pigmentation and freckling in European populations [60,61]. Variants in the *LYST* gene have been associated with eye color in a Dutch sample [53]. Finally, polymorphisms in the genes *EGFR* and *OPRM1* have been recently associated with skin pigmentation in admixed samples from the New World [49]. We provide more detailed information about each gene, its relevance in the pigmentation pathway, and a description of previous natural selection scans or association studies with pigmentary phenotypes, if relevant, as Additional file 3.

**Table 1:** Pigmentation genes that are outliers based on the empirical distribution of different tests of positive selection

** Gene**	** iHS**	** LSBL**	** Rsb-Eas-Afr**	**Rsb-Eas-Eur**	** Tajima D**	** H**	D*	F*
**Top 0.1%**								
*EDAR*	+(0.0019, 2)	++ (9.3E-06, 5)	++ (9.6E-04, 2)	+(0.0049, 1)	++(6.2E-04, 3)			
					CHB			
*MITF*					++ (9.9E-04, 4)	+(0.0037, 3)	++ (2.1E-04, 5)	++ (1.8E-04, 6)
					CHB/CHS/JPT	CHB/CHS/JPT	CHS/JPT	CHS/JPT
*ATRN*					+(0.0077, 2)		++ (9.6E-04, 3)	++ (9.4E-04, 6)
					CHB		CHB	CHB/CHS
*OCA2*		++(5.6E-05, 5)						
*TRPM1*			+(0.0012, 6)	+(0.0019, 5)	++(8.9E-04, 1)			
					CHS			
*TH*	++(7.9E-04, 1)		+(0.0073, 1)	+(0.0028, 1)				
*KLHL7*							++(8.81E-04, 5)	+(0.0010, 5)
							CHB/CHS	CHB/CHS
*TMEM33*					++(1.72E-04, 9)		+(0.0029, 3)	+(0.0017, 3)
					CHB/CHS/JPT		CHB	CHB
**Top 1%**								
*PMEL (SILV)*							+(0.0051, 2)	+(0.0072, 2)
							CHS	CHS
*BNC2*					+ (0.0026, 2)	+ (0.0056, 2)	+ (0.0051, 1)	+ (0.0039, 1)
					CHB/CHS	CHS/JPT	CHS	CHS
*EGFR*		+ (0.0029, 3)	+ (0.0056, 1)	+ (0.0028, 1)				
*LYST*			+ (0.0019, 1)				+ (0.0072, 1)	+ (0.0057, 1)
							CHS	CHS
*DCT*		+ (0.0099, 1)					+(0.0064, 1)	+(0.0067, 1)
							CHB	CHB
*OPRM1*					+ (0.0079, 1)			
					JPT			
*TYRP1*						+(0.0081, 1)		
						CHS		
*MC1R*	+(0.0073, 1)		+ (0.0027, 1)					
*MLPH*			+(0.0015, 4)			+(0.0014, 3)		
						CHB/CHS/JPT		
*ADAM17*			+(0.0015, 2)	+(0.0044, 2)	+(0.0021, 3)			
					JPT			
*CTSD*							+(0.0045, 1)	+(0.0044, 1)
							JPT	JPT
*PDIA6*			+(0.0069, 1)			+(0.0072, 1)		
						JPT		

The analysis was based on windows of 25 kilobases. In each cell, we indicate if the gene is in the top 0.1% (++) or 1% (+) of the empirical distribution for the relevant statistics. In parenthesis, we report the smallest p-value observed for any of the windows overlapping the gene, and the number of windows with p-values < 0.01. For the Site Frequency Spectrum tests (D, H, D*, and F*) we also indicate the East Asian populations in which we identified outlier 25 kb windows.

**Figure 1:**
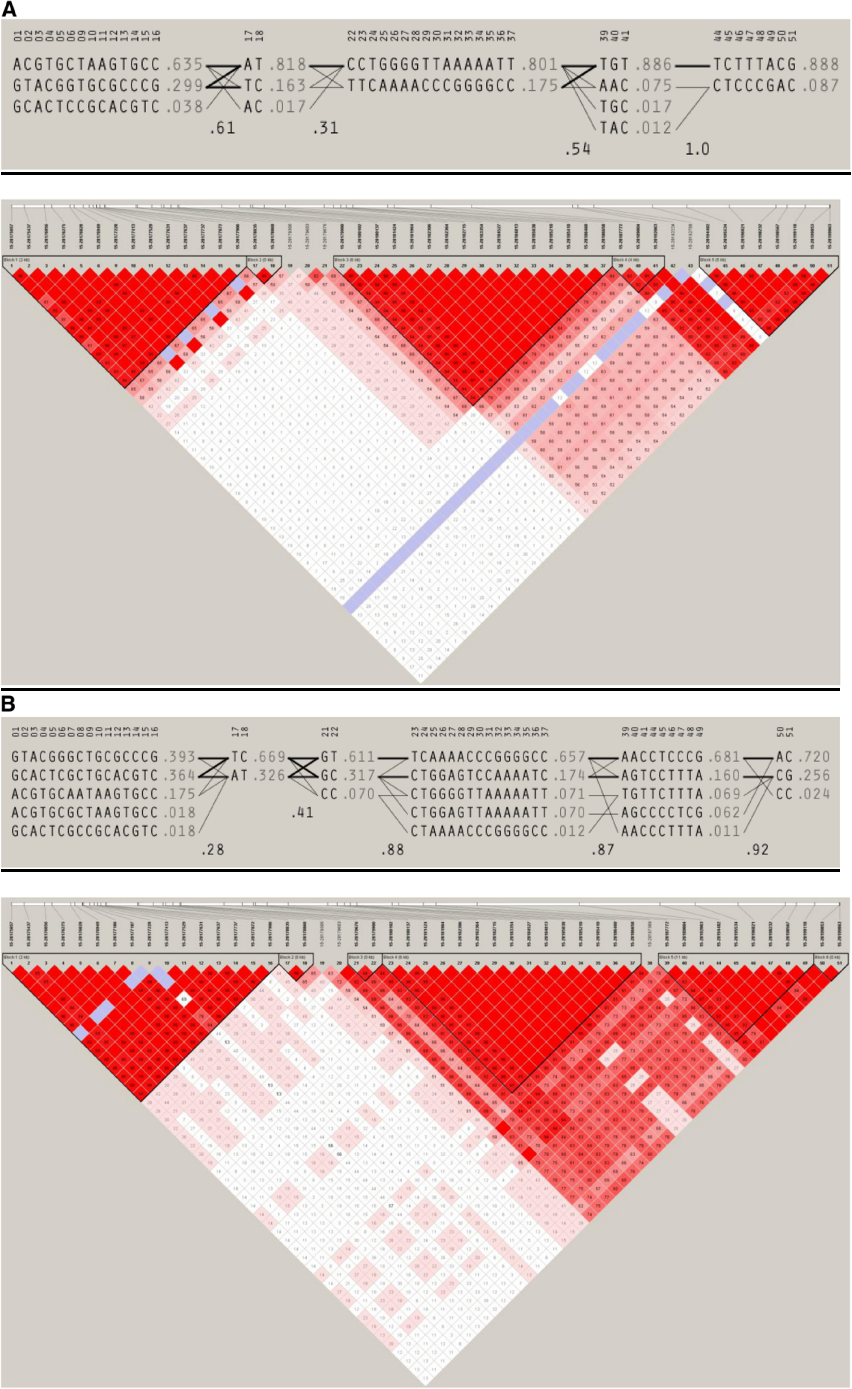
Haplotype block structure of OCA2 gene (window chr15:28,175,001-28,200,000) A. In East Asian 1000 Genomes samples.B. In European 1000 Genomes samples.

One of the methods employed in our study (LSBL) was designed to highlight genomic regions showing extreme differentiation in one population, with respect to other groups. In this study, we were primarily interested in identifying genomic regions that differentiate East Asian populations with respect to Europeans and Africans. In particular, we would like to find regions in which positive selection may have driven the reduction of melanin levels specifically in East Asia. In order to explore this in more detail, we used the program Haploview [62] to compare the haplotype structure of the candidate pigmentation genes showing large LSBL values in East Asians (*EDAR, OCA2, EGFR* and *DCT*) with the haplotype structure observed in European populations, which are also characterized by reduced melanin content. For the top LSBL windows found for each of these genomic regions, we identified overlapping common markers (frequency higher than 1%) between the East Asian and European 1000 Genomes reference samples, and used the program Haploview to generate the haplotype block structure in each population, using the default algorithm [63]. As expected, we observed very large differences in haplotype frequencies in these regions between the East Asian and European populations. These haplotype differences range between 59% (*DCT*) and 90% (*EDAR*) and these contrasting patterns indicate that positive selection may have favored specific haplotypes in East Asian populations. Figure 1 shows the haplotype structure observed for the *OCA2* gene in East Asian and European populations. The largest differences in frequency are observed for East Asian haplotype blocks 4 and 5, which span slightly more than 10 kilobases, from position 28,187,772 to 28,199,863 on chromosome 15. Interestingly, the non-synonymous variant that has been associated with skin pigmentation in East Asians, rs1800414 [23,29] is located within this region (genomic position 28,197,037 in Genome Built 37.3). Other studies have also reported distinct signatures of positive selection and different haplotype distributions for OCA2 in Europe and East Asia [12,14,24,64,65]. The haplotype structure of the genes *EDAR*, *DCT*, and *EGFR* in European and East Asian populations is provided as Additional file 4. These analyses confirm that some genes relevant for pigmentation biology show extreme haplotype differentiation between European and East Asian populations, and suggest that a careful analysis of haplotype variation, in combination with a detailed annotation of the variants present in the relevant windows, may help to identify the genetic variants responsible for the selective sweeps in East Asians.

In summary, we have carried out a genome-wide analysis of selection signatures in East Asian populations, focusing on genes that are relevant for pigmentation biology. This analysis allowed us to identify a number of genes that show extremely unusual patterns of genetic variation in East Asia. It is in principle possible that some of these findings are false positives and are not due to the action of recent positive selection in East Asian populations. However, most of these genes are outliers for different tests and/or different populations (CHB, CHS, JPT), and have been described in previous scans for positive selection, providing strong support to the hypothesis that recent selective sweeps left a signature in these regions. It is important to note that, even if selective sweeps are responsible for these unusual patterns of variation, it is possible that the selective factors involved did not have any effect on melanin levels in East Asian populations. Many of these genes are expressed widely and have a broad range of functions, and these selective sweeps may be related to phenotypes other than pigmentation. For example, certain mutations of the Ectodysplasia A receptor gene *(EDAR)*, which is an extreme outlier based on three different types of test, are associated with pigmentary phenotypes in mice (http://www.informatics.jax.org/), and for this reason *EDAR* is a pigmentation candidate gene. However, this gene is also important in the development of hair, teeth, and other ectodermal derivatives, and mutations in this gene have been associated with several traits in humans, including hypohidrotic ectodermal dysplasia [66] shovel-shaped incisors [67] and hair thickness [68]. In a recent study [69], researchers generated a knock-in mouse to test the phenotypic consequences of the EDARV370A (370A) polymorphism, which has been associated with hair thickness and incisor shoveling in East Asian populations. The researchers found that 370A homozygous mice had thicker hair than 370V homozygous mice, similarly to the patterns observed in human populations. Importantly, they also observed that the 370A mice had smaller mammary fat pads and increased eccrine gland numbers. An association study in individuals of Han descent showed that the 370A allele was associated with shoveling of the upper incisors and also eccrine gland density. These findings suggest that the dramatic increase in the frequency of the 370A allele in East Asia could have been driven by selection favoring more efficient evapo-transpiration, although the authors also mentioned the possibility that reduced mammary fat pad size could also have been adaptive, or, given the clear pleiotropic effects of the 370A mutation, that selection acted on multiple traits. This fascinating example highlights the challenges encountered when trying to explain the ultimate selective factors responsible for some of the selective sweeps observed in human populations, and emphasizes the importance of association studies, functional studies, and studies in animal models to complement genome-wide scans of selection signatures.

## Conclusions

Our study has identified a list of genes that could potentially explain the reduction of melanin levels that took place in East Asia after the out-of-Africa migration of anatomically modern humans. The application of recently developed methods, such as the Composite of Multiple Signals (CMS) test [42] or Approximate Bayesian Computation (ABC) tests [70], and a more extensive annotation of the polymorphisms present within and around these genes, may be useful to narrow down the genic regions that were the target of positive selection, and to distinguish between selection that has acted on newly arisen mutations or standing variation. However, it will be necessary to carry out association studies in samples for which quantitative data on pigmentary traits are available, and functional studies in melanocytes to confirm the implication of these genes in normal pigmentation variation.

## Abbreviations

HGDP: Human Genome Diversity Project; LD: Linkage Disequilibrium; SNP: Single Nucleotide Polymorphism; JPT: Japanese; CHB: Han Chinese from Beijing; CHS: Southern Han Chinese; LSBL: Locus Specific Branch Length; iHS: Integrated Haplotype Score; SFS: Site Frequency Spectrum; DAVID: Database for Annotation, Visualization and Integrated Discovery; CMS: Composite of Multiple Signals; ABC: Approximate Bayesian Computation.

## Competing interests

The authors have declared that no competing interests exist.

## Authors' contributions

JLH participated in acquisition of data, contributed to the analysis and interpretation of data, and wrote the first draft of the manuscript, TS, RG, AR and JMA contributed to the analysis and interpretation of data, ME contributed to the acquisition of data, EJP was responsible for study conception and design, contributed to the analysis and interpretation of data and participated in the preparation of the final version of the manuscript. All authors read and approved the final version of the manuscript.

## Supplementary Material

Additional file 1: Table S1 List of the top 0.1% windows for all the statistics used in this study. Click here for file

Additional file 2: Table S2 Basic annotation of the genes overlapping the top 0.1% windows identified in this study. The annotations were obtained with the DAVID database. The table reports the gene, the gene name, chromosome location, statistical tests for which the gene is an outlier, other references that have reported signatures of selection in the relevant genes, disease class for which genetic associations have been reported, OMIM_disease, KEGG pathway and GOTERM Biological Pathway. Click here for file

Additional file 3 Description of the pigmentation genes identified in the scan for signatures of selection in East Asia. Click here for file

Additional file 4 Haplotype block structure of the genes DCT, EDAR and EFGR in the East Asian and European 1000 Genomes samples. Click here for file
