# Supplementary material for: Exploring signatures of positive selection in pigmentation candidate genes in populations of East Asian ancestry
Source: BMC Evol Biol. 2013 Jul 12;13:150. doi: 10.1186/1471-2148-13-150 (PMC3727976; doi:10.1186/1471-2148-13-150)
Supplement: Additional file 3 — Description of the pigmentation genes identified in the scan for signatures of selection in East Asia. [file 1471-2148-13-150-S3.docx]

***ADDITIONAL FILE 3.***

Description of the pigmentation genes identified in the scan for signatures of selection in East Asia.

1. **Extreme outliers (top 0.1% of the empirical distribution), in alphabetic order.**

***ATRN (Attractin)***- This gene encodes attractin, a type 1 membrane protein that acts as an accessory receptor for the Agouti protein (*ASIP*). Agouti antagonizes *αMSH* at *MC1R* (a key receptor in pigment synthesis), promoting pheomelanin production. When *αMSH* binds to *MC1R*, eumelanin is produced; consequently *ATRN* plays a role in the switch between these two pigment types [1]. *ATRN* is responsible for the *mahogany* mutation (*mg)* found in mice. Animals with the null mutation in *ATRN* produce only eumelanin and thus have darker coats in addition to tremors, sprawling gate and abnormal neural morphology [2]. Several studies have reported evidence of signatures of selection for this gene in East Asian populations, using different types of statistics [3-5].

***EDAR (Ectodysplasia A receptor)* -** This gene encodes a receptor for Ectodysplasin A, a member of the tumor necrosis factor receptor family (*TNFR*). It is required for the development of hair, teeth, and other ectodermal derivatives. Mutations in this gene are associated with pigmentary phenotypes in mice (<http://www.informatics.jax.org/>). In humans, mutations in *EDAR* have been associated with hypohidrotic ectodermal dysplasia [6], shovel-shaped incisors [7], hair thickness [8] and eccrine gland density [9]. Numerous studies have reported evidence of a selective sweep in this gene in East Asian populations [10-13].

***KLHL7 (Kelch-like protein 7)***- The protein encoded by this gene may be involved in protein degradation. Mutations in this gene have been linked with retinitis pigmentosa 42. This disorder happens due to a decrease in ligase activity and an accumulation of molecules set for proteasomal break down [14]. This results in the accretion of dark pigment and damage to the retina.

***MITF (Microphthalmia-associated transcription factor)-***This gene encodes a helix loop helix-leucine zipper protein that is involved in cell development, including the development of neural crest-derived melanocytes and optic retinal pigment epithelial cells [15]. MITF increases eumelanin synthesis through the activation of tyrosinase. It is also involved in melanocyte differentiation. In humans, mutations in *MITF* cause Waardenburg syndrome type2a (OMIM#193510). This syndrome is characterized by abnormal pigmentation and deafness, as a result of anomalies in cell differentiation [16]. Similar disturbances are found in mice with the *microphtalmia* (*mi*) mutations, which show early-onset deafness and loss of pigmentation in the eye and skin (feet, tail, ears) [15]. *MITF* has also been associated with graying with age and vitiligo [17]. The SNP rs149617956 has been associated with melanoma in Australians [18]. McEvoy et al. (2006) [3], Myles et al. (2007) [19] and Norton et al. (2007) [4] reported evidence of positive selection in the *MITF* gene.

***OCA2 (Oculocutaneous albinism 2, also called the P gene)-*** The gene *OCA2* encodes a transmembrane protein involved in small molecule transport in melanosomes, which is important in melanogenesis. OCA2 may also regulate melanosomal pH, which affects pigmentation [22]. Additionally, it regulates tyrosinase (TYR), which is the key enzyme driving melanin synthesis [23]. *OCA2* is associated with the most frequent form of albinism (oculocutaneous albinism type 2, OMIM#203200). Variants in this gene have also been associated with normal variation in skin and iris pigmentation. Variants located in the nearby *HERC2* gene, which affect the transcription of the *OCA2* gene, are strongly associated with blue eye color in European populations [24-29]. Another variant, which is common in East Asian populations but absent or very rare in Europe, has been associated with skin pigmentation in East Asia [30-31]. Polymorphisms in the *OCA2* gene have also been associated with vitiligo in Europeans [32]. Deletions of the *OCA2* gene are often associated with Prader-Willi and Angelman syndromes while extra copies result in generalized hyperpigmentation of the skin [33]. *OCA2* causes *pink eye dilution* (p) in mice. The p gene was one of the earliest known coat mutations in mice. At least 12 mutations in the *p* gene are known and affect pigmentation, behavior and development in mice [34]. Putative signals of selection have been reported in numerous studies for this gene, including McEvoy et al. (2006) [3], Voight et al. (2006) [35], Izagirre et al. (2006) [36], Duffy et al. (2007) [33], Lao et al. (2007) [37], Norton et al. (2007) [4] and Donnelly et al. (2012) [38].

***TH (Tyrosine hydroxylase)****-* The protein encoded by this gene may be involved in the hydroxylation of tyrosine to L-Dopa in melanocytes, which is a key step in melanin synthesis. Marles et al. (2003) [39] found tyrosine hydroxylase isoform I in the cytosol and melanosomes of melanocytes. These authors suggested that in the melanosomes this protein may promote pigmentation in concert with tyrosinase. Gillbro et al., (2004) [40] also demonstrated the presence of TH in melanocytes as cytoplasmic granules.

***TMEM33 (Transmembrane protein 33)-***The protein encoded by this gene is a multi-pass membrane protein. It has been identified in melanosome fractions from stage I to stage IV, using mass spectrometry [41]. Putative selective signatures in *TMEM33* have been described by Pickrell et al, (2009) [13] and Zhong et al, (2010) [42].

***TRPM1*** ***(Transient receptor potential channel, subfamily M, member 1)-***The protein encoded by this gene is known to interact with MITF [43]. *TRPM1* is expressed in melanocytes and retinal cells. Further, dark-pigmented skin showed higher content of TRPM1 in melanocytes than light-pigmented skin [44]. This gene has been linked with the spotting of the skin in Appaloosa horses, with decreased expression in Appaloosa (*LP*) versus non-LP horses [45]. Polymorphisms within this gene in the bear homologue (*MLSN1*) may be associated with pelage colors [46]. Williamson et al. (2007) [5] reported putative signatures of selection for this gene in East Asians.

1. **Other outliers (top 1% of the empirical distribution), in alphabetic order.**

***ADAM17 (ADAM metalloproteinase domain 17)-*** This gene encodes a protein involved in development [4]. ADAM17 activates molecules such as TNF*,* TGF (transforming growth factor) and NOTCH1, which may be important in melanocyte development. Mice homozygous for mutations in this gene generally died peri-natally. Those that survived showed eye degeneration, perturbed coats, curly vibrissae and irregular patterns of pigmentation [47]. In 2005, Horiuchi et al. [48] described that mice deficient for ADAM17 had densely pigmented hair follicles and disorganized pigment granules in the hair. Population-specific selection signatures have been reported for this gene in East Asians [3, 4, 13].

***BNC2 (Basonuclin 2)-***This gene encodes a transcription factor that may play an important role in skin keratinocytes and melanocytes. It is believed to maintain proliferative capacity and prevent terminal differentiation*.* A recent study reported that variants in *BNC2* were associated with skin pigmentation in Europeans [49]. This gene has also been associated with freckling in a GWAS study in Northern Europeans [50]. Some mutations in the homologous genes in mice and zebrafish are also associated with pigmentary phenotypes [51, 52]. McEvoy et al, (2006) [3] have reported putative signals of positive selection in this gene.

***CTSD*** ***(Cathepsin D)*** - This gene encodes a lysosomal aspartyl protease that is widely expressed and is involved in proteolytic degradation, apoptosis and cell invasion. This protein was over-represented in mice with an exfoliation syndrome caused by a *LYST* mutation, in which iris pigment dispersion and transillumination defects occur. Thus, CTSD may play a role in the LYST pathway and consequently, pigmentation [53]. Follo et al (2011) [54] reported that a knockout in Zebrafish caused hyperpigmentation in addition to abnormal retinal pigment. Cathepsin D has been identified by mass spectrometry in melanosome fractions from stage I to stage IV [55].

***DCT (Dopachrome tautomerase)-*** The protein encoded by this gene is an important component of the tyrosinase enzyme complex (*TYR, TRP1, DCT*) and is found on the melanosome membrane. It is responsible for the conversion of tyrosine into melanin and the regulation of eumelanin and pheomelanin synthesis. Knockout mice had lighter coats as a result of a decrease in dopachrome-tautomerase activity [56]. Typically, mutations in this gene increase pheomelanin synthesis and decrease eumelanin synthesis [57]. Myles et al., (2006) [19] and Lao et al. (2007) [37] described evidence of selection signatures in this gene, primarily in East Asians.

***EGFR (Epidermal growth factor receptor)-***This gene encodes a receptor for members of the epidermal growth factor family. EGFR may have important functions in keratinocyte proliferation, differentiation, migration and death [58]. In turn, keratinocytes regulate the proliferation as well as the differentiation of melanocytes. This pathway is thought to be the mechanism for the *Dsk5* mutation in mice, which causes a darkening of footpads as a result of an increased number of melanocytes and the proliferation of basal keratinocytes [59]. This gene has been recently associated with skin pigmentation in admixed samples from the New World. Signatures of positive selection in this gene have been reported by Akey et al. (2002) [60], Lao et al. (2007) [37] and Quillen et al. (2011) [61], including putative signals in East Asian samples.

***LYST (Lysosomal trafficking regulator)-*** The protein encoded by this gene is involved in organelle biosynthesis and size regulation [41]. In humans, mutations in this gene are responsible for Chediak-Higashi syndrome (CHS, OMIM#214500), which is characterized by decreased pigmentation of hair and eyes, among other traits [62]. Mutations in mice cause the *beige* phenotype (bg). This mutant has giant melanosomes (typically resulting in coat dilution) [41]. *LYST* is known to interact with *RAB27A,* another pigmentation candidate. A mutation resulting in grey coat color and seizures has also been noted. Melanosomes of the hair follicle, choroid eye layer and neural tube-derived pigment epithelium of the retina were large and irregular in shape and the secretory vesicles of the dermal mast cells were also enlarged [63]. Studies of cattle coat color showed that *LYST* may affect the intensity of pigment, with no resulting coat color changes. A GWAS study in Dutch has reported that two SNPs within the *LYST* gene were associated with iris saturation [27]. McEvoy et al. (2007) [3] and Norton et al. (2007) [4] reported signatures of selection for this gene, including signals in East Asians.

***MC1R (Melanocortin 1 receptor gene)***- The protein encoded by this gene is a member of the family of G protein-coupled receptors known as melanocortin receptors and has a critical role in switching between eumelanin and pheomelanin synthesis in the melanocytes. Binding of the melanocyte-stimulating hormone (α–MSH) to MC1R results in activation of adenylyl cyclase, and increased levels of intracellular cAMP and tyrosinase activity. The ultimate outcome is the production of eumelanin within the melanocytes. Conversely, when the antagonist agouti signaling protein (ASIP) binds to MC1R, there is a decrease in tyrosinase activity and a switch to pheomelanin production. Several polymorphisms in the *MC1R* gene have a strong association with red hair/fair skin phenotype in European populations (Asp84Glu, Arg151Cys, Arg160Trp and Asp294His), and other variants also show a weak association with these traits (Val60Leu, Val92Met and Arg163Gln) [64]. The derived 163Gln allele, which is present in very high frequencies in East Asian populations (>60%), but very low frequencies in European and African populations, has been recently associated with lighter skin in an East Asian sample [65]. The *MC1R* gene has also been associated with cutaneous malignant melanoma and UV-induced skin damage (OMIM# 155555). Coop et al. [66] reported evidence of positive selection for this gene in East Asian populations.

***MLPH (Melanophillin)-*** The protein encoded by this gene is a rab effector protein, involved in the migration of melanosomes. Mature melanosomes are transported in melanocytes through microtubules. They reach the cell periphery and are then captured in the cortical actin network. They are then transferred to keratinocytes. The genes *RAB27A, MYO5A* and *MLPH* code for some of the key proteins involved in this process, known as a tripartite complex, which act as a tether for melanosomes to bind the actin network. RAB27A recruits its downstream effector, MLPH, which binds to actin and to MYO5A. When any of these proteins fails, the transfer of melanosomes to keratinocytes does not occur, and the melanosomes will be localized perinuclearly [20]. This results in hypopigmentation, as dendrites are lacking in pigment. In humans, mutations in these proteins lead to Griscelli syndrome, type 3 (OMIM#609227) [21]. In mice the mutation is termed *leaden* (*ln*). In these mutant mice, melanin becomes clumped in large masses and this can cause a dilution of coat color. Myles et al. (2007) [19] and Pickrell et al. (2009) [13] reported the presence of signatures of selection in the *MLPH* region.

***OPRM1(Opioid receptor, mu 1)-***This gene encodes an opioid receptor. Its function as a neuronal regulator suggests an association with skin color due to the common roots of the nervous system and skin. It is now known that opioid receptors are functional in keratinocytes and melanocytes and may also have an effect on their differentiation. The underlying mechanism is currently unknown. Quillen et al. (2011) [61] reported that variants in the *OPRM1* were associated with skin pigmentation in admixed samples from the New World. These authors also reported that *OPRM1* showed evidence of selection signatures in indigenous American populations.

***PDIA6*** ***(Protein disulfide isomerase family A, member 6)-*** This gene belongs to a family of isomerases that catalyze formation, reduction, and isomerization of disulfide bonds in proteins and are thought to play a role in folding of disulfide-bonded proteins [67]. PDIA6 has been found in melanosome fractions from stage I to IV [55]. Based on FST statistics, Ortiz et al. [68] indicated that this gene shows suggestive signatures of selection in humans.

***PMEL*** ***(Premelanosome protein, also known as SILV)-*** This gene encodes a melanocyte-specific type I transmembrane glycoprotein that plays an key role in the structural organization of premelanosomes. In dogs, mutations within this gene have been associated with the merle coat pattern, which is characterized by patches of diluted pigment (OMIM# 155550). In mice, a mutation in this gene is responsible for the *silver* phenotype, characterized by varying intensities of silvering. In humans, Frudakis et al. reported association of *PMEL (SILV)* haplotypes with iris color [69]. Using the Composite Likelihood Ratio (CLR) test, Williamson et al. [5] reported evidence of positive selection for this gene in East Asian populations.

***TYRP1*** ***(Tyrosinase related protein 1)-*** The protein encoded by this gene is a melanosomal enzyme that belongs to the tyrosinase family. TYRP1 plays an important role in melanin synthesis. Mutations in this gene are responsible for oculocutaneous albinism III in humans (OCA3, OMIM#203290) and other pigmentary phenotypes in mice and cats (OMIM# 115501). Polymorphisms in the *TYRP1* gene have been associated with hair and iris color in European populations [70], and a non-synonymous mutation that is only found in Oceania was recently associated with blond hair in Melanesians [71]. Santos et al. [72] reported that *TYRP1* showed strong evidence of positive selection in East Asians, based on FST and EHH tests.

**REFERENCES**

1. Hubbard JK, Uy AC, Hauber ME, Hoekstra HE, and Safran R: **Vertebrate Pigmentation: from underlying genes to adaptive function.** *Trends in Genetics* 2010, **5**: 231-239.

# 2. Hida T, Wakamatsu K, Sviderskaya EV, Donkin AJ, Montoliu L, Lamoreux ML, Yu B, Millhauser GL, Ito Shosuke, Barsh GS, Jimbow K, Bennett DC. Agouti protein, mahogunin, and attractin in pheomelanogenesis and melanoblast-like alteration of melanocytes: a cAMP-independent pathway. *Pigment Cell Melanoma Res* 2009, 22: 623–634.

3. McEvoy B, Beleza S, Shriver MD: **The genetic architecture of normal variation in human pigmentation: an evolutionary perspective and model**. *Hum Mol Genet 2006*, **15** (suppl 2):176–181.

4. Norton H et al: **Genetic evidence for convergent evolution of light skin in European and East Asians**. *Mol Bio Evol* 2007, **24**: 710-722.

5. Williamson SH, Hubisz MJ, Clark AG, Payseur BA, Bustamante CD, Payseur BA, Bustamante CD, Nielsen R**: Localizing recent adaptive evolution in the human genome.** *PLoS Genet* 2007, **3**: e90.

6. Cluzeau C, Hadj-Rabia S, Jambou M, Mansour S., Guigue P, Masmoudi S, Bal E, Chassaing N, Vincent MC, Viot G, Clauss F, Maniere MC, Toupenay S, Le Merrer M, Lyonnet S, Cormier-Daire V, Amiel J, Faivre L, de Prost Y, Munnich A, Bonnefont JP, Bodemer C, Smahi A: **Only four genes (EDA1, EDAR, EDARADD, and WNT10A) account for 90% of hypohidrotic/anhidrotic ectodermal dysplasia cases**. *Hum Mutat* 2011, **32**: 70–72.

7. Kimura R, Yamaguchi T, Takeda M, Kondo O, Toma T, Hanejk K, Hanihara T, Matsukusa H, Kawamura S, Maki K, Osawa M, Ishida H, Oota H: **A common variation in EDAR is a genetic determinant of shovel-shaped incisors**. *Am J Hum Genet* 2009, **85**:528-535.

8. Fujimoto A, Kimura R, Ohashi J, Omi K, Yuliwulandari R, Batubara L, Mustofa MS, Samakkarn U, Settheetham-Ishida W, Ishida T: **A scan for genetic determinants of human hair morphology: EDAR is associated with Asian hair thickness**. *Hum Mol Genet* 2008: **17**:835–843.

9. Kamberov YG, Wang S, Tan J, Gerbault P, Wark A, Tan L, Yang Y, Li S, Tang K, Chen H, Powell A, Itan Y, Fuller D, Lohmueller J, Mao J, Schachar A, Paymer M, Hostetter E, Byrne E, Burnett M, McMahon AP, Thomas MG, Lieberman DE, Jin L, Tabin CJ, Morgan BA, Sabeti PC: **Modeling recent human evolution in mice by expression of a selected EDAR variant**. *Cell* 2013*,* **152**: 691–702.

10. Kelley JL, Madeoy J, Calhoun JC, Swanson W, Akey JM: **Genomic signatures of positive selection in humans and the limits of outlier approaches**. *Genome Res* 2006, **16**: 980–989.

11. Kimura R, Fujimoto A, Tokunaga K, Ohashi J: **A Practical Genome Scan for Population-Specific Strong Selective Sweeps That Have Reached Fixation**. *PLoS ONE* 2007, **2**: e286

12. Sabeti PC, Varilly P, Fry B, Lohmueller J, Elizabeth Hostetter E, Cotsapas C, Xie X, Byrne EH, McCarroll SA, Gaudet R, Schaffner SF, Lander ES &[The International HapMap Consortium](http://www.nature.com/nature/journal/v449/n7164/full/nature06250.html#The-International-HapMap-Consortium): **Genome-wide detection and characterization of positive selection in human populations**. *Nature* 2007, **449**:913-918.

# 13. Pickrell JK, Coop G, Novembre J, Kudaravalli S, Li JZ, Absher D, Srinivasan BS, Barsh GS, Meyers RM, Feldman MW, Pritchard JK: Signals of recent positive selection in a worldwide sample of human populations*. Genome Res* 2009, 19:826-837.

# 14. Kigoshi Y, Tsuruta F, Chiba T: Ubiquitin ligase activity of Cul3-KLHL7 protein is attenuated by autosomal dominant retinitis pigmentosa causative mutation. *J Biol Chem* 2011, 38:33613-21.

15. Fuse N, Yasumoto K, Takeda K, Amae S, Yoshizawa M, Udono T, Takahashi K, Tamai M, Tomita Y, Tachibana M, Shibahara S: **Molecular cloning of cDNA encoding a novel microphthalmia-associated transcription factor isoform with a distinct amino-terminus.** *J. Biochem* 1999, **126**: 1043-1051.

16. Tachibana M, Takeda K, Nobukuni Y, Urabe K, Long JE, Meyers KA, Aaronson SA, Miki T: **Ectopic expression of MITF, a gene for Waardenburg syndrome type 2, converts fibroblasts to cells with melanocytes characteristics.** *Nature Genet*. **14:** 50-54, 1996

17. Montoliu L, Oetting WS, Bennett DC: **Color Genes*.*** *European Society for Pigment Cell Research.* World Wide Web (February, 2013) (URL: http://www.espcr.org/micemut).

18. Yokoyama S, Woods SL, Boyle GM, Aoude LG, MacGregor S, Zismann V, Gartside M, Cust AE, Haq R, Harland M, Taylor JC, Duffy DL, Holohan K, Dutton-Regester K, Palmer JM, Bonazzi V, Stark MS, Symmons J, Law MH, Schmidt C, Lanagan C, O'Connor L, Holland EA, Schmid H, Maskiell JA, Jetann J, Ferguson M, Jenkins MA, Kefford RF, Giles GG : **A novel recurrent mutation in MITF predisposes to familial and sporadic melanoma.** *Nature* 2011, **480**: 99-103.

19. Myles S, Somel M, Tang K, Kelso J, Stoneking M: **Identifying genes underlying skin pigmentation differences among human populations**. *Human Genetics* 2007, **120**: 613-621.

# 20. Westbroek W, Klar A, Cullinane AR, Ziegler SG, Hurvitz H, Ganem A, Wilson K, Dorward H, Huizing M, Tamimi H, Vainshtein I, Berkun Y, Lavie M, Gahl WA, Anikster Y: Cellular and clinical report of new Griscelli syndrome type III cases. *Pigment Cell Melanoma Research* 2011, 25:47-56.

21. Sanal O, Ersoy F, Tezcan I, Metin A, Yel L, Menasche G, Gurgey A, Berkel I, de Saint Basile G. **Griscelli disease: genotype-phenotype correlation in an array of clinical heterogeneity.** J. Clin. Immun. 22: 237-243, 2002.

22. Scherer D and Kumar R: **Genetics of pigmentation in skin cancer**. *Mutat Res* 2010, **705**: 141-153.

23. Pan T, Zhu J, Hwu W-J, Jankovic J: **The Role of Alpha-Synuclein in Melanin Synthesis in Melanoma and Dopaminergic Neuronal Cells**. *PLoS ONE* 2012, **9**: e45183.

24. Sulem P, Gudbjartsson DF, Stacey SN, Helgason A, Rafnar T, Magnusson KP, Manolescu A, Karason A, Palsson A, Thorleifsson G, Jakobsdottir M, Steinberg S, Palsson S, Jonasson F, Sigurgeirsson B, Thorisdottir K, Ragnarsson R, Benediktsdottir KR, Aben KK, Kiemeney LA, Olafsson JH, Gulcher J, Kong A, Thorsteinsdottir U, Stefansson K: **Genetic determinants of hair, eye and skin pigmentation in Europeans**. Nature Genetics 2007, **39**:1443-1452.

25. Kayser M, Liu F, Janssens AC, Rivadeneira F, Lao O, van Duijn K, Vermeulen M, Arp P, Jhamai MM, van Ijcken WF, Den Dunnen JT, Heath S, Zelenika D, Despriet DD, Klaver CC, Vingerling JR, De Jong PT, Hofman A, Aulchenko YS, Uitterlinden AG, Oostra BA, Van Duijn CM: **Three genome-wide association studies and a linkage analysis identify *HERC2* as a human iris color gene**. *Am J Hum Genet* 2008, **82**:411–423.

26. Sturm RA, Duffy DL, Zhao ZZ, Leite FPN, Stark MS Hayward NK, Martin NG, Montgomery GW: **A single SNP in an evolutionary conserved region within intron 86 of the HERC2 gene determines human blue-brown eye color**. *Am J Hum Genet* 2008, **82**: 424–431

27. Liu F, Wollstein A, Hysi PG, Ankra-Badu GA, Spector TD, Park D, Zhu G, Larsson M, Duffy DL, Montgomery GW Mackey DA, Walsh S, Lao O, Hofman A, Rivadeneira F, Vingerling JR, Uitterlinden AG, Martin NG, Hammond CJ, Kayser M: **Digital quantification of human eye color highlights genetic association of three new loci**. *PLoS Genet* 2010, **6**:e1000934.

28. Cook AL, Chen W, Thurber AE, Smit DJ, Smith AG, Bladen TG, Brown DL, Duffy DL,

Pastorino L, Bianchi-Scarra G, Leonard JH, Stow JL, Sturm RA: **Analysis of cultured human melanocytes based on polymorphisms within the SLC45A2/MATP, SLC24A5/NCKX5, and OCA2/P loci.** *J Invest Dermatol* 2009, **129**: 392–405.

29. Visser M, Kayser M, Palstra RJ: **HERC2 rs12913832 modulates human pigmentation by attenuating chromatin-loop formation between a long-range enhancer and the OCA2 promoter**. *Genome Research* 2012, **22**: 446–455.

30. Edwards M, Bigham A, Tan J, Li S, Gozdzik A, Ross K, Jin L, Parra EJ: **Association of the *OCA2* Polymorphism His615Arg with Melanin Content in East Asian Populations: Further Evidence of Convergent Evolution of Skin Pigmentation**. *PLoS Genet* 2010, 6: e1000897.

31. Abe Y, Tamiya G, Nakamura T, Hozumi Y, Suzuki T: **Association of melanogenesis genes with skin color variation among Japanese females**. *J Dermatol Sci* 2013, **69**:167-172.

32. Ying J, Birlea SA, Fain PR, Ferrara TM, Ben S, Riccardi SL, Cole JB, Gowan K, Holland PJ, Bennett DC, Luiten RM, Wolkerstorfer A. Van der Veen WJP, Hartmann A, Elchner S, Schuler G, van Geel N, Lambert J, Kemp EH, Gawkrodger DJ, Weetman AP, Taieb A, Jouary T, Ezzedine K, Wallace MR, McCormack WT, Picardo M, Leone G, Overbeck A, Silverberg NB, and Spritz RA: **Genome-wide association analyses identify 13 new susceptibility loci for generalized vitiligo**. *Nature Genetics* 2012, **44**:676-680.

33. Duffy DL, Montgomery GM, Chen W, Zhao ZZ, Le L, James MR, Hayward NK, Martin NG, Sturm RA: **A Three–Single-Nucleotide Polymorphism Haplotype in Intron 1 of *OCA2* Explains Most Human Eye-Color Variation**. *Am J Hum Genet* 2007, **80**: 241–252.

34. Lyon MF, King TR, Gondo Y, Gardner JM, Nakatsu Y, Eicher EM, Brilliant MH: **Genetic and molecular analysis of recessive alleles at the pink-eyed dilution (*p*) locus of the mouse.** *Proc Natl Acad Sci USA* 1992; **89**:6968–6972.

# 35. Voight BF, Kudaravalli S, Wen X, Pritchard JK: A map of recent positive selection in the human genome*. PLoS Biol* 2006, 4: e72

36. Izagirre N, Garcia I, Junquera, de la Rua C, Alonso S: **A scan for signatures of positive selection in candidate loci for skin pigmentation in humans**. *Mol Biol Evol* 2006, **23**:1697-1706

37. Lao O, De Gruijter JM, Van Duijn K, Navarro A, Kayser M: **Signatures of positive selection in genes associated with human skin pigmentation as revealed from analyses of single nucleotide polymorphisms.** *Ann Hum Genet* 2007, **71**:354-369.

# 38. Donnelly MP, Paschou P, Grigorenko E, Gurwitz D, Barta C, Lu RB, Zhukova OV, Kim JJ, Siniscalco M, New M, Li H, Kajuna SL, Manolopoulos VG, Speed WC, Pakstis AJ, Kidd JR, Kidd KK: A global view of the OCA2-HERC2 region and pigmentation.

*Hum Gen* 2012, **13**: 683-96.

39. Marles LK, Peters EM, Tobin DJ, Hibberts NA and Schallreute KU: **Tyrosine hydroxylase isoenzyme I is present in human melanosomes: a possible novel function in pigmentation**. *Experimental Dermatology* 2003, **12**: 61–70.

40. Gillbro JM, Marles LK, Hibberts NA, Schallreuter KU: **Autocrine catecholamine biosynthesis and the beta-adrenoceptor signal promote pigmentation in human epidermal melanocytes**. *J Invest Dermatol* 2004, **123**: 346–353.

**41. Chi A, Valencia JC, Hu ZZ, Watabe H, Yamaguchi H, Mangini NJ, Huang H, Canfield VA, Cheng KC, Yang F, Abe R, Yamagishi S, Shabanowitz J, Hearing VJ, Wu C, Appella E, Hunt DF: Proteomic and bioinformatic characterization of the biogenesis and function of melanosomes. *J Proteome Res* 2006, 5:3135-3144.**

42. Zhong M, Lange K, Papp JC, Fan R: **A powerful score test to detect positive selection in genome-wide scans**. *Europ J Hum Genet* 2010, **18**:1148–1159.

43. Hunter JJ, Shao J, Smutko JS, Dussault BJ, Nagle DL, Woolf EA, Holmgren LM, Moore KJ, Shyjan AW: **Chromosomal localization and genomic characterization of the mouse melastatin gene (Mlsn1).** *Genomics* 1998, **54**: 116-123.

44. Oancea E, Vriens J, Brauchi S, Jun J, Splawski I, Clapham DE: **TRMP1** **forms ion channels associated with melanin content in melanocytes.** *Sci Signal* 2009, **2**: ra21ra21.

45. Bellone, RR, Brooks SA, Sandmeyer L, Murphy BA, Forsyth G, Archer S, Bailey E, Grahn B: **Differential gene expression of TRPM1, the potential cause of congenital stationary night blindness and coat spotting patterns (LP) in the Appaloosa horse (Equus caballus).** *Genetics* 2008, **179**: 1861-1870.

46. Miller, W, Schuster SC, Welch AJ, Ratan A, Bedoya-Reina OC, Zhao F, Kim HL, Burhans RC, Drautz DI, Wittekindt NE, Tomsho LP, Ibarra-Laclette E, Herrera-Estrella L, Peacock E, Farley S, Sage GK, Rode K, Obbard M, Montiel R, Bachmann L, Ingólfsson O, Aars J, Mailund T, Wiig Ø, Talbot SL, Lindqvist C: **Polar and brown bear genomes reveal ancient admixture and demographic footprints of past climate change**. *Proc Natl Acad Sci USA* 2012, **109**:E2382–E2390.

47. Peschon JJ, Slack JL, Reddy P, Stocking KL, Sunnarborg SW, Lee DC, Russell WE, Castner BJ, Johnson RS, Fitzner JN, Boyce RW, Nelson N, Kozlosky CJ, Wolfson MF, Rauch CT, Cerretti DP, Paxton RJ, March CJ, Black RA: **An essential role for ectodomain shedding in mammalian development**. *Science* 1998, **5392**:1281–1284.

48. Horiuchi K, Zhou HM, Kelly K, Manova K, Blobel CP: **Evaluation of the contributions of ADAMs 9, 12, 15, 17, and 19 to heart development and ectodomain shedding of neuregulins beta1 and beta2**. *Dev Biol* 2005, **283**: 459-471.

49. Jacobs LC, Wollstein A, Lao O, Hofman A, Klaver CC, Uitterlinden AG, Nijsten T, Kayser M, Liu F: **Comprehensive candidate gene study highlights UGT1A and BNC2 as new genes determining continuous skin color variation in Europeans**. *Hum Genet* 2013, **132**:147–158.

50. Eriksson N, Macpherson JM , Tung JY, Hon LS, Naughton B, Saxonov S, Avey L, Wojcicki A, Pe'er I, Mountain J: [**Web-Based, Participant-Driven Studies Yield Novel Genetic Associations for Common Traits**](http://www.ncbi.nlm.nih.gov/pmc/articles/PMC2891811/)**.** *PLoS Genet* 2010, **6**: e1000993.

51. Javerzat S and Jackson IJ: **White-based brown (*Tyrp1B*^−^*w*) is a dominant mutation causing reduced hair pigmentation owing to a chromosomal inversion**. *Mamm Genome* 1998, **9**:469–471.

52. Lang MR, Patterson LB, Gordon TN, Johnson SL, Parichy DM: ***Basonuclin-2*Requirements for Zebrafish Adult Pigment Pattern Development and Female Fertility.** *PLoS Genet* 2009*,* **11**: e1000744.

53. Trantow CM, Cuffy TL, Fingert JH, Kuehn MH, Anderson MG: **Microarray analysis of iris gene expression in mice with mutations influencing pigmentation.** *Invest Ophthalmol Vis Sci.* 2011, **52**:237–248.

54. Follo C, Ozzano M, Mugoni V, Castino R, Santoro M and Isidoro C: **Knock-down of cathepsin D affects the retinal pigment epithelium, impairs swim-bladder ontogenesis and causes premature death in zebrafish**. *PLoS ONE* 2011, **6**:e21908

55. Dennis G, Sherman BT, Hosack DA, Yang J, Baseler MW, Lane HC, Lempicki RA. DAVID: **Database for Annotation, Visualization, and Integrated Discovery**. *Genome Biol* 2003, **4**: P3. Epub.

56. Jackson IJ, Chambers DM, Tsukamoto K, Copeland NG, Gilbert DJ, Jenkins NA, Hearing V: **A second tyrosinase-related protein, TRP-2, maps to and is mutated at the mouse slaty locus**. *EMBO J* 1992; **11**:527–535.

57. Costin GE, Valencia JC, Wakamatsu K, Ito S, Solano F, Milac AL, Vieira WD, Yamaguchi Y, Rouzaud F, Petrescu AJ, et al: **Mutations in dopachrome tautomerase (Dct) affect eumelanin/pheomelanin synthesis, but do not affect intracellular trafficking of the mutant protein**. *Biochem J* 2005, **59**:391:249.

58. Jost M, Kari C, Rodeck U: **EGF receptor-an essential regulator of multiple epidermal function**. *Eur Jour Dermat* 2000, **10**: 505-510.

59. Fitch KR, McGowan KA, van Raamsdonk CD, Fuchs H, Lee D, Puech A, Herault Y, Threadgill DW, Hrabe de Angelis M, Barsh GS: **Genetics of dark skin in mice**. *Genes & Dev* 2003, **17**:214–228.

60. Akey JM, Zhang G, Zhang K, Jin L, and Shriver MD: **Interrogating a High-Density SNP Map for Signatures of Natural Selection.** *Genome Res* 2002, **12**:1805-1814.

61. Quillen EE, Bauchet M, Bigham AW, Delgado-Burbano ME, Faust FX, Klimentidis YC, Mao X, Stoneking M, Shriver MD: **OPRM1 and EGFR contribute to skin pigmentation differences between Indigenous Americans and Europeans.** *Hum Genet* 2011, **13**:1073–1080.

62. Spritz RA: **Multi-organellar disorders of pigmentation: tied up in traffic**. *Clin Genet* 1999, **55:** 309-317.

63. Runkel F, Bussow H, Seburn KL, Cox GA, Ward DM, Kaplan J, Franz T: **Grey, a novel mutation in the murine LYST gene, causes the beige phenotype by skipping of exon 25.** *Mammalian Genome* 2006*,* **17**: 203-210.

64. Duffy D, Box N, Chen W, Palmer JS, Montgomery GW, James MR, Hayward NK, Martin NG, Sturm RA: **Interactive effects of MC1R and OCA2 on melanoma risk phenotypes**. *Hum Mol Genet* 2004, **13**:447-461.

65. Yamaguchi K, Watanabe C, Kawaguchi A, Sato T, Naka I, Shindo M, Moromizato K, Aoki K, Ishida H, Kimura R: **Association of melanocortin 1 receptor gene (MC1R) polymorphisms with skin reflectance and freckles in Japanese**. *J Hum Genet* 2012, **57**:700-708.

66. Coop G, Pickrell JK, Novembre J, Kudaravalli S, Li J, et al: **The Role of Geography in Human Adaptation**. *PLoS Genet* 2009, **5**: e1000500.

67. Kikuchi, M., Doi, E., Tsujimoto, I., Horibe, T., Tsujimoto, Y: **Functional analysis of human P5, a protein disulfide isomerase homologue.** *J Biochem* 2002, **132:** 451-455.

68. Ortiz M, Guex N, Patin E, Martin O, Xenarios I, Ciuffi A, Quintana-Murci L, Telenti A: **Evolutionary trajectories of primate genes involved in HIV pathogenesis**. Mol Biol Evol 2009, 26: 2865-2875.

69. Frudakis T, Thomas M, Gaskin Z, Venkateswarlu K, Suresh Chandra K, Ginjupalli S, Gunturi S, Natrajan S, Ponnuswamy VK, Ponnuswamy KN: **Sequences associated with human iris pigmentation**. *Genetics* 2003, **165**: 2071-2083.

70. Sulem P, Gudbjartsson DF, Stacey SN, Helgason A, Rafnar T, Jakobsdottir M, Steinberg S, Gudjonsson SA, Palsson A, Thorleifsson G, Pálsson S, Sigurgeirsson B, Thorisdottir K, Ragnarsson R, Benediktsdotttir KR, Aben KK, Vermeulen SH, Goldstein AM, Tucker MA, Kiemeney LA, Olafsson JH, Gulcher J, Kong A, Thorsteinsdottir U, Stefansson K: **Two newly identified genetic determinants of pigmentation in Europeans*.*** *Nat Genet* 2008, **40**:835-837.

71. Kenny EE, Timpson NJ, Sikora M, Yee MC, Moreno-Estrada A, Eng C, Huntsman S, Burchard EG, Stoneking M, Bustamante CD, Myles S: **Melanesian blond hair is caused by an amino acid change in TYRP1***. Science* 2012, **336**:554.

**72. Alonso S, Izagirre N, Smith-Zubiaga I, Gardeazabal J, Díaz-Ramón JL, Díaz-Pérez JL, Zelenika D, Boyano MD, Smit N and de la Rúa C: Complex signatures of selection for the melanogenic loci TYR, TYRP1 and DCT in humans**. *BMC Evol Biol* 2008, **8:** 1471-2148.
