# Supplementary material for: Exploring signatures of positive selection in pigmentation candidate genes in populations of East Asian ancestry
Source: BMC Evol Biol. 2013 Jul 12;13:150. doi: 10.1186/1471-2148-13-150 (PMC3727976; doi:10.1186/1471-2148-13-150)

**ADDITIONAL FILE 4**

Haplotype block structure of the genes *DCT*, *EDAR* and *EFGR* in the East Asian and European 1000 Genomes samples.

Figure S1: Haplotype block structure of the *DCT* gene (window chr13:95,075,001-95,100,000) A/ In East Asian 1000 Genomes samples. B/ In European 1000 Genomes samples.

A/


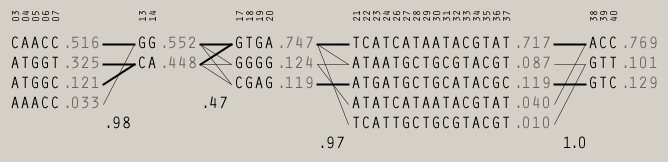


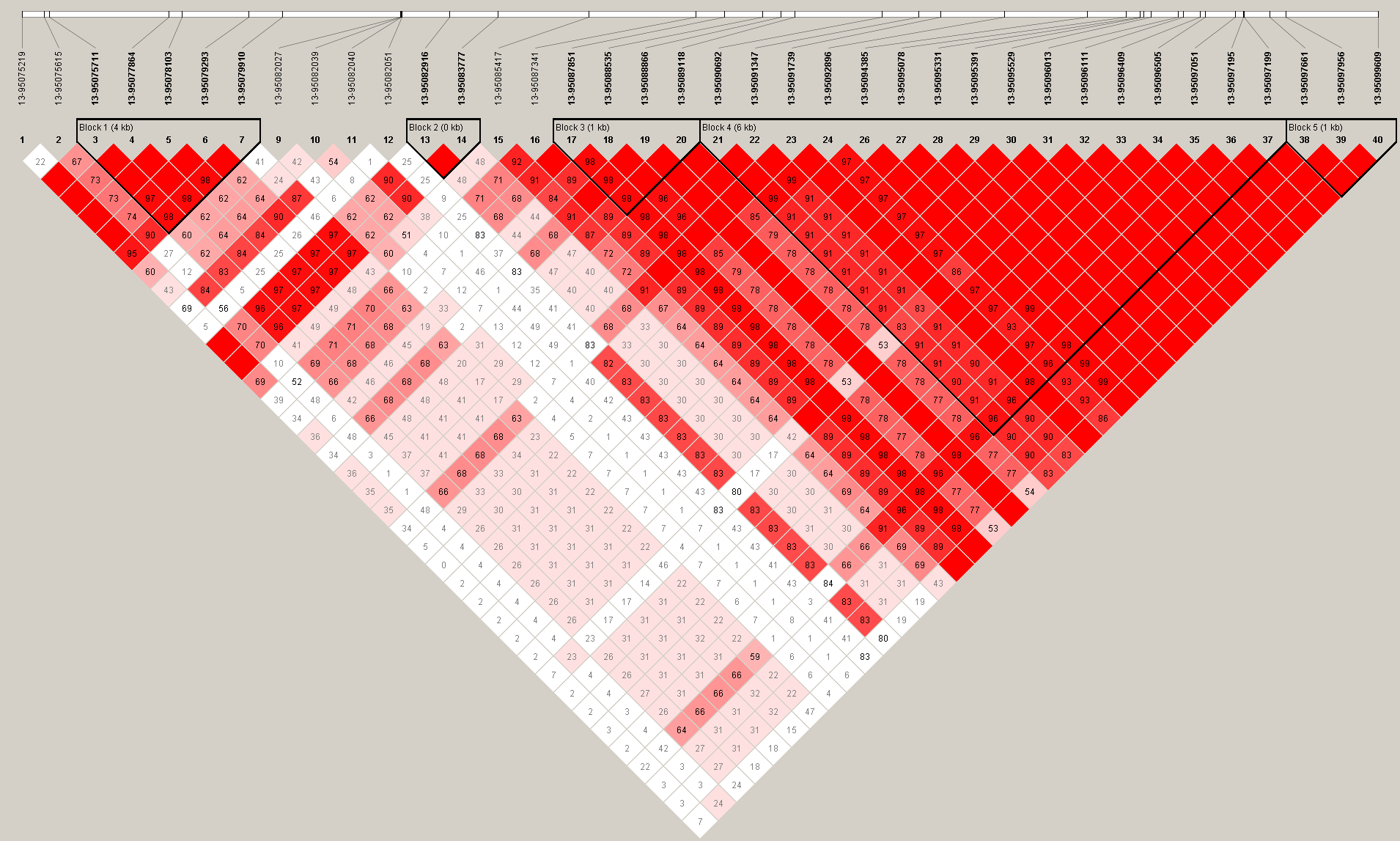


B/


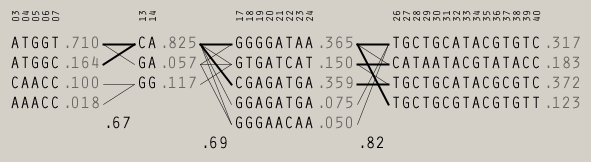


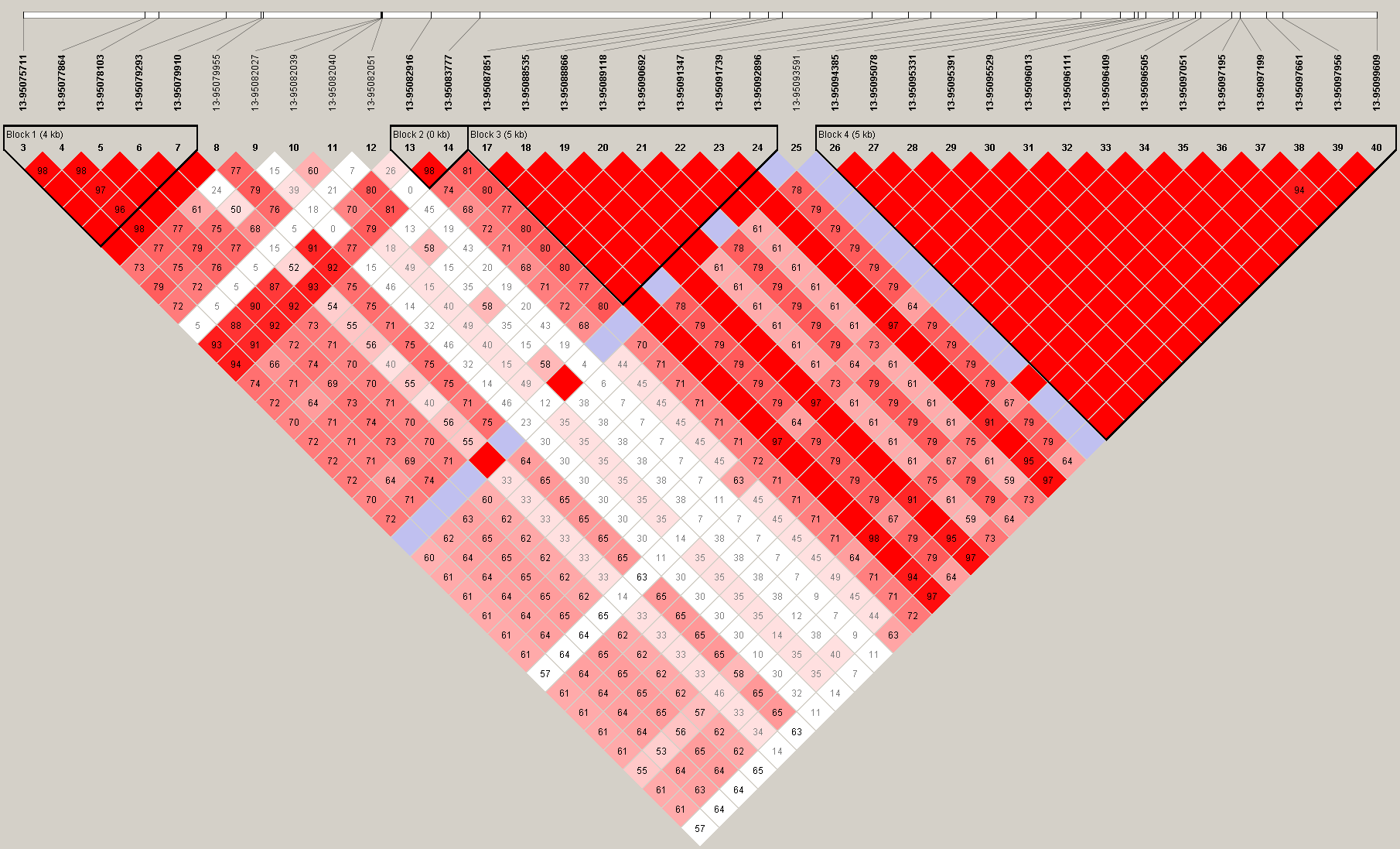


Figure S2: Haplotype block structure of the *EDAR* gene (window chr2:109,500,001-109,550,000) A/ In East Asian 1000 Genomes samples. B/ In European 1000 Genomes samples.

A/


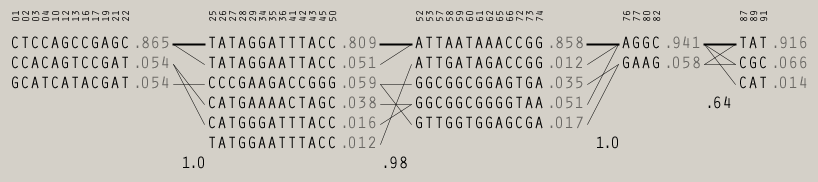


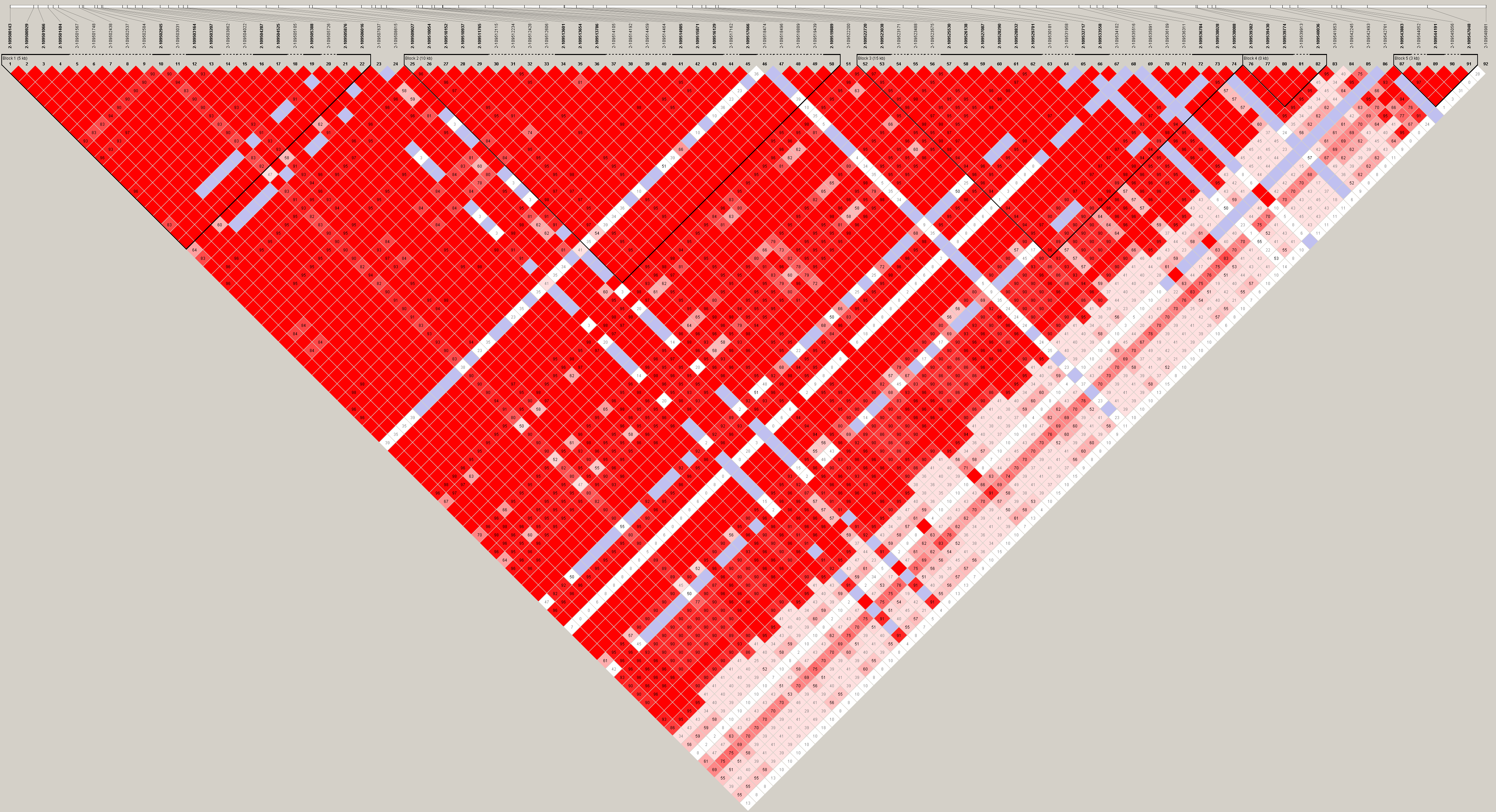


B/


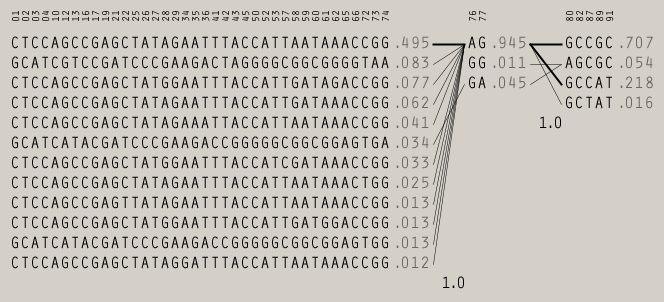


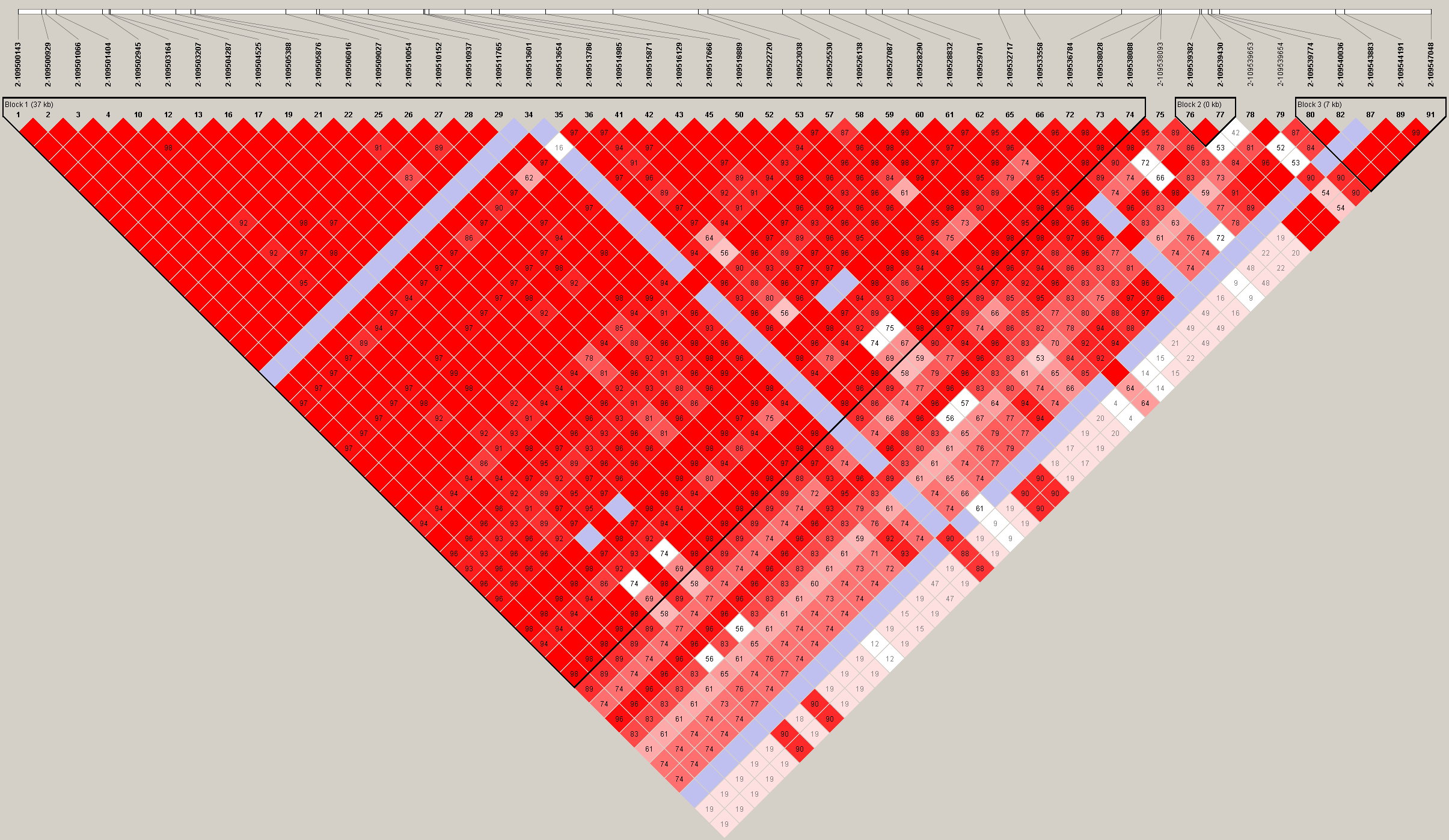


Figure S3: Haplotype block structure of the *EFGR* gene (window chr7:55,125,001-55,175,000) A/ In East Asian 1000 Genomes samples. B/ In European 1000 Genomes samples.

A/


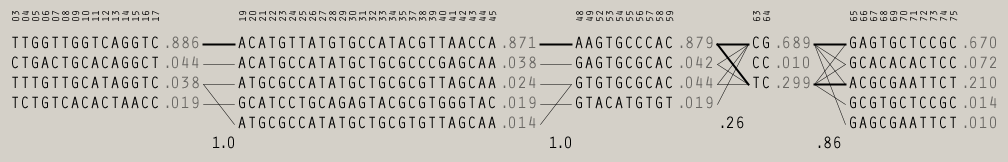


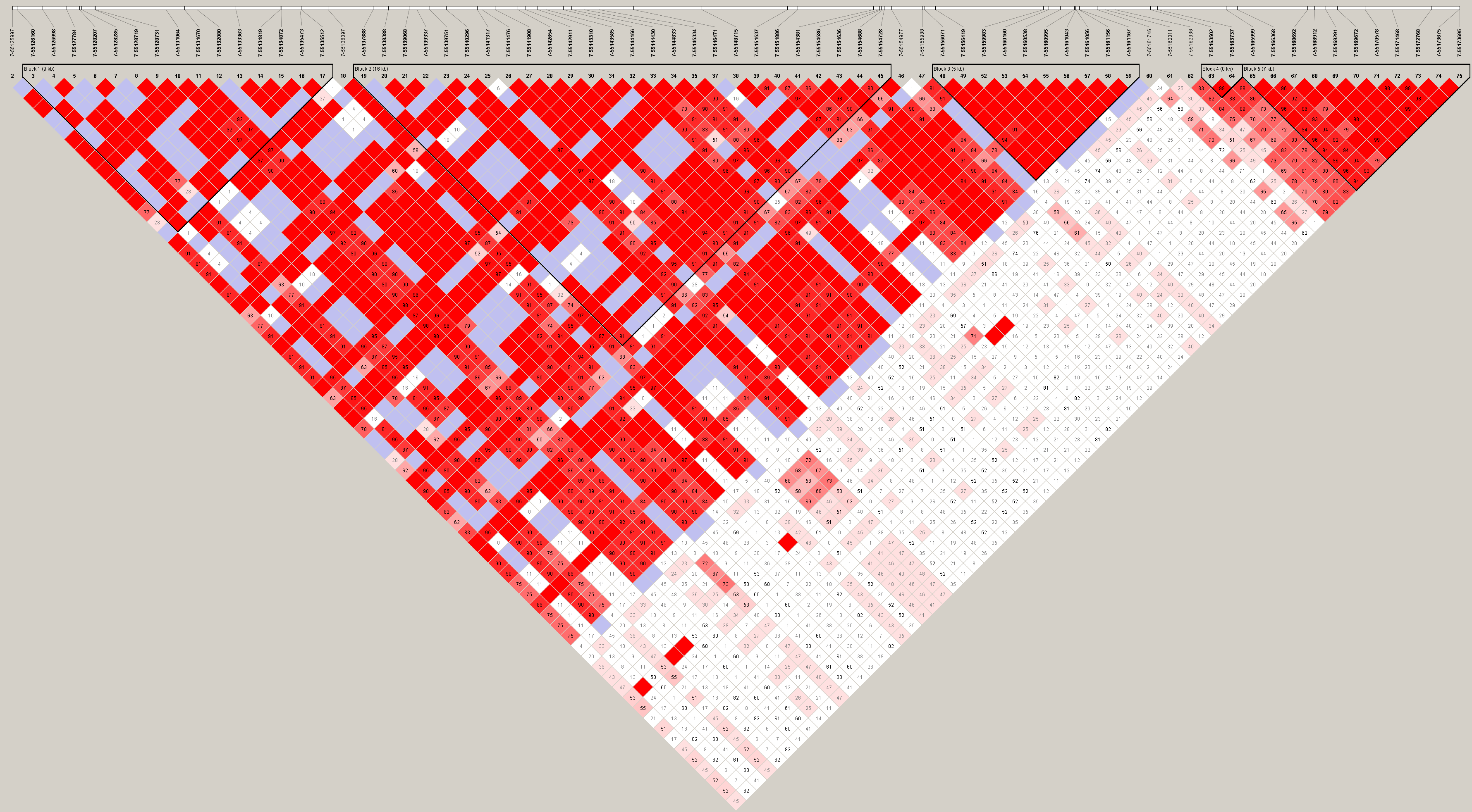


B/


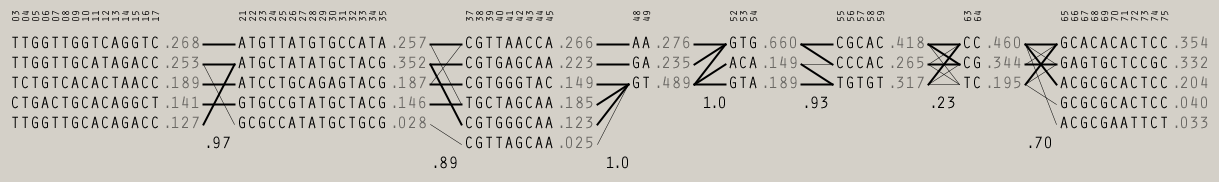


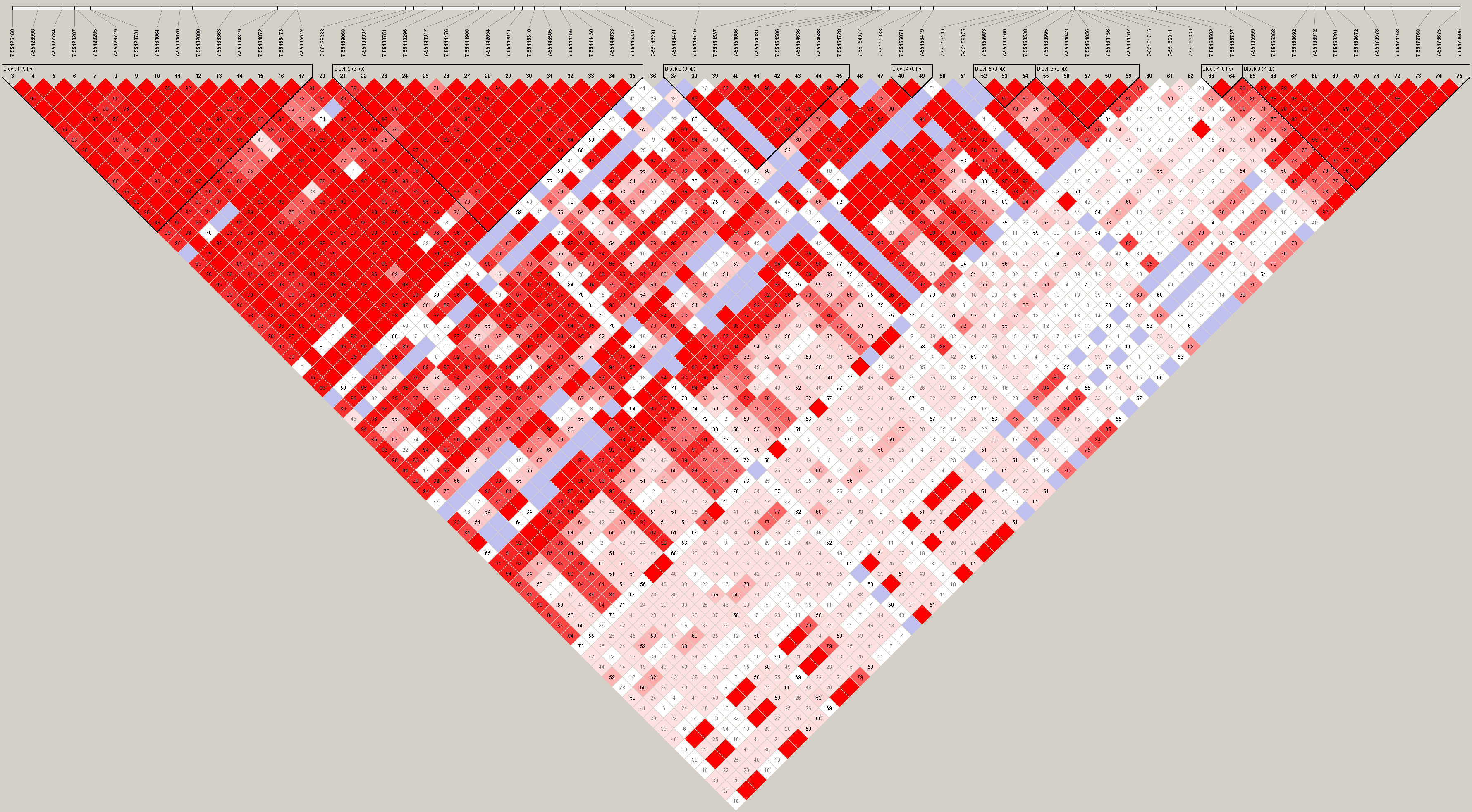

Supplement: Additional file 4 — Haplotype block structure of the genes DCT, EDAR and EFGR in the East Asian and European 1000 Genomes samples. [file 1471-2148-13-150-S4.docx]
